# Strengthening African health systems through global health diplomacy

**DOI:** 10.34172/hpp.2020.45

**Published:** 2020-11-07

**Authors:** Vijay Kumar Chattu, Shalini Pooransingh

**Affiliations:** ^1^Division of Occupational Medicine, Department of Medicine, Faculty of Medicine, University of Toronto, Toronto, ON, M5S 1A8, Canada; ^2^Institute of International Relations, The University of the West Indies, St. Augustine, Trinidad and Tobago; ^3^Department of Paraclinical Sciences, Faculty of Medical Sciences, The University of the West Indies, St. Augustine, Trinidad and Tobago

## Dear Editor,

Nepomnyashchiy et al^1^ in their recent *Lancet* publication highlight thedeficiencies in African health systems for handling COVID-19. They discuss human resources, non-availability of supplies and emphasize on building resilient health systems with community health workers at the grassroot level. According to the World Health Organization (WHO), Africa accounts for a quarter of the global mortality and morbidity burdens of communicable and non-communicable diseases but its share of global health expenditure is less than 1%, leaving more than half of its population without access to essential health services.^2^ To their credit the Africa Centers for Disease Control and Prevention (CDC), a technical institution of the African Union, was established in 2016 and launched in 2017 with the aim of supporting Member States and their Public Health Institutes in detecting and responding to public health threats.

We would argue that more than grassroot level activity is required to tackle diseases like COVID-19. Public health specialists encounter barriers in sending and receiving timely health information.^3^ Since global health diplomacy brings together the disciplines of public health, foreign policy, economics, security, international affairs etc.^4^ It would be prudent for the Africa CDC and others to embrace global health diplomacy to strengthen their capacity for disease preparedness and response. One approach is to consider the role of Health Attachés in Embassies and Foreign Missions. These professionals can inspire a new model for international partnerships and multilateral health security networks which could early detect health trends and monitor indicators well in advance of a new disease spreading beyond control, benefiting all involved nations.

## Competing interests

The authors declare that they have no competing interests.

## Ethical approval

Not applicable.

## Authors’ contributions

VKC prepared the initial draft and SP edited the draft. Both VKC and SP approved the final version.
